# Bone Remodeling and the Role of TRAF3 in Osteoclastic Bone Resorption

**DOI:** 10.3389/fimmu.2018.02263

**Published:** 2018-09-28

**Authors:** Brendan F. Boyce, Jinbo Li, Lianping Xing, Zhenqiang Yao

**Affiliations:** Department of Pathology and Laboratory Medicine, Center for Musculoskeletal Research, University of Rochester Medical Center, Rochester, NY, United States

**Keywords:** osteoclast, osteoblast, NF-kappaB, RANK, TNF, TRAF3, TRAF6

## Abstract

Skeletal health is maintained by bone remodeling, a process in which microscopic sites of effete or damaged bone are degraded on bone surfaces by osteoclasts and subsequently replaced by new bone, which is laid down by osteoblasts. This normal process can be disturbed in a variety of pathologic processes, including localized or generalized inflammation, metabolic and endocrine disorders, primary and metastatic cancers, and during aging as a result of low-grade chronic inflammation. Osteoclast formation and activity are promoted by factors, including cytokines, hormones, growth factors, and free radicals, and require expression of macrophage-colony stimulating factor (M-CSF) and receptor activator of NF-κB ligand (RANKL) by accessory cells in the bone marrow, including osteoblastic and immune cells. Expression of TNF receptor-associated factor 6 (TRAF6) is required in osteoclast precursors to mediate RANKL-induced activation of NF-κB, which is also necessary for osteoclast formation and activity. TRAF3, in contrast is not required for osteoclast formation, but it limits RANKL-induced osteoclast formation by promoting proteasomal degradation of NF-κB-inducing kinase in a complex with TRAF2 and cellular inhibitor of apoptosis proteins (cIAP). TRAF3 also limits osteoclast formation induced by TNF, which mediates inflammation and joint destruction in inflammatory diseases, including rheumatoid arthritis. Chloroquine and hydroxychloroquine, anti-inflammatory drugs used to treat rheumatoid arthritis, prevent TRAF3 degradation in osteoclast precursors and inhibit osteoclast formation *in vitro*. Chloroquine also inhibits bone destruction induced by ovariectomy and parathyroid hormone in mice *in vivo*. Mice genetically engineered to have TRAF3 deleted in osteoclast precursors and macrophages develop early onset osteoporosis, inflammation in multiple tissues, infections, and tumors, indicating that TRAF3 suppresses inflammation and tumors in myeloid cells. Mice with TRAF3 conditionally deleted in mesenchymal cells also develop early onset osteoporosis due to a combination of increased osteoclast formation and reduced osteoblast formation. TRAF3 protein levels decrease in bone and bone marrow during aging in mice and humans. Development of drugs to prevent TRAF3 degradation in immune and bone cells could be a novel therapeutic approach to prevent or reduce bone loss and the incidence of several common diseases associated with aging.

## Introduction

The skeleton provides support for propulsion by skeletal muscles as well as vital protection for internal organs, including the brain and heart. It is also a repository for calcium and other elements that get deposited in bone as it mineralizes during bone formation and are released from bone when it is being remodeled. In this way, bone participates in the control of calcium levels in the blood and tissues (1) to mediate numerous cellular functions, including contraction of skeletal and cardiac muscles (2). Bone remodeling is a normal physiological process that maintains skeletal integrity after skeletal development by removing small foci of damaged or effete bone from bone surfaces and replacing them with new bone (3, 4). By this mechanism, the skeleton is continuously renewed throughout life.

**Figure 1:**
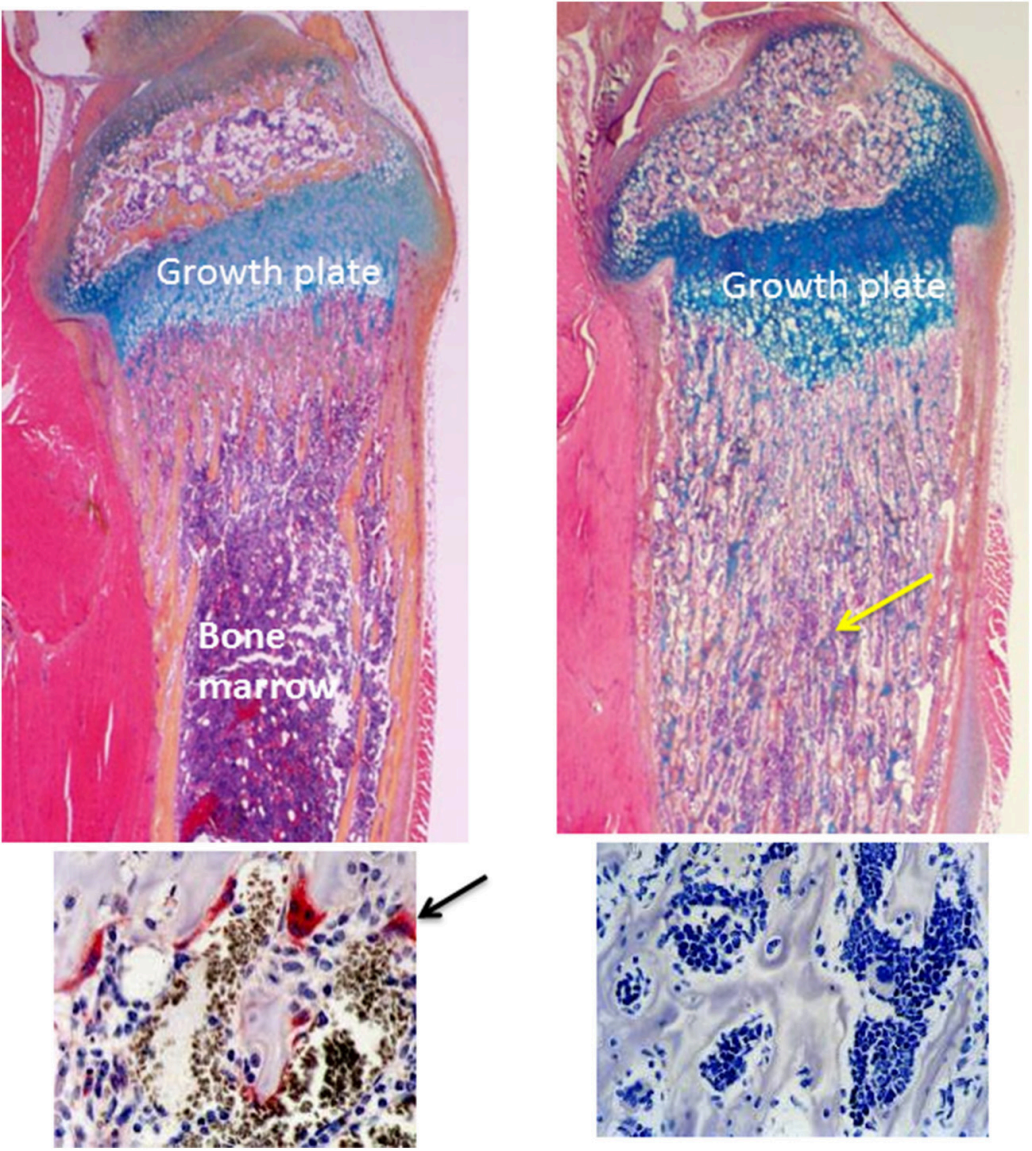
Normal and osteopetrotic tibial bones from wild-type and RANK^−/−^ mice. The upper panels are H&E-stained longitudinal sections of tibiae from 4-weeks-old mice showing a normal growth plate and underlying metaphyseal trabecular bone and bone marrow from a wild-type mouse (left panel) and a thickened growth plate and unremodeled osteopetrotic bone (yellow arrow) filling the medullary cavity from a RANK^−/−^ mouse (right panel). The lower panels are TRAP-stained sections of the bone beneath the growth plate showing TRAP-positive (red, arrow) osteoclasts in the wild-type tibia and absence of osteoclasts and TRAP staining in the RANK^−/−^ tibia.

During embryonic development, bone is formed by osteoblasts, specialized mesenchyme-derived cells that lay down layers (lamellae) of matrix composed of mainly type 1 collagen (3, 5), which is mineralized a few days later. Numerous other non-collagenous proteins are also deposited in the bone matrix, including osteocalcin, sialoproteins, glycoproteins, proteoglycans, TGFβ, bone morphogenetic proteins (BMPs) and fibroblast growth factors (FGFs) (6, 7). These proteins and minerals are released from bone during bone resorption and in increased amounts in numerous pathologic processes in which bone destruction is elevated. They can influence the behavior of cells in the bone microenvironment and outside the skeleton, particularly in pathologic processes in which remodeling is increased (5, 8, 9). During development, long bones are formed initially in cartilage molds roughly in the shape that the bones will have before birth (3). The cartilage is resorbed by TRAP-positive osteoclasts, but it is also removed by chondroclasts, poorly characterized tartrate–resistant acid phosphatase (TRAP)-negative cells that perform this function in mice in the absence of RANKL or RANK expression (10, 11). This is based on remodeling and removal of the much of the cartilage in bone metaphyses beneath growth plates and its replacement by bone in the absence of TRAP-positive cells in RANKL^−/−^ and RANK^−/−^ (Figure 1) mice (10, 11). Growth plates form at the proximal and distal ends of embryonic long bones. These plates consist of columns of small resting proliferating chondrocytes and larger hypertrophic chondrocytes, which lay down matrix that calcifies at the interface between them and the bone marrow (3). This calcified matrix is resorbed and replaced by bone, which also is calcified. Hypertrophic chondrocytes express RANKL to attract osteoclast precursors from adjacent sinusoids in the marrow (10), and like osteoblastic cells, they also express osteoprotegerin (OPG) (12, 13), a decoy receptor for RANKL, that prevents RANKL binding to RANK to limit osteoclast formation (4, 10). 1, 25 dihydroxy Vitamin D3, BMP2 and Wnt/β-catenin signaling proteins also are expressed by these chondrocytes in which they regulate expression of RANKL (10).

In the adult skeleton, bone remodeling begins with removal of microscopic foci of calcified bone matrix by osteoclasts, which form trenches on bone surfaces, called resorption lacunae. Osteoclast formation requires expression of M-CSF and RANKL by accessory cells in the bone microenvironment and of their receptors by osteoclast precursors (4, 14). Signaling downstream from these receptors regulates the differentiation of osteoclast precursors into osteoclasts as well as the resorptive activity and survival of osteoclasts. The M-CSF receptor is a tyrosine kinase that phosphorylates and activates downstream signaling molecules (15). In contrast, RANK does not possess kinase activity and recruits TRAFs, which are adaptor proteins that form complexes that activate mitogen-activated protein kinases (MAPKs), NF-κB and activator protein-1 (AP-1) signaling (16). TRAFs play important positive and negative regulatory roles in RANKL-induced osteoclast formation and activation (16, 17) in normal bone remodeling and in many pathologic processes affecting the skeleton in which bones can weaken to the point where they can fracture readily. This review will briefly describe the mechanisms that regulate bone remodeling, with emphasis on osteoclast formation in normal and pathologic processes, and the roles that TRAFs play in osteoclast and osteoblast formation and function, focusing on the evolving roles of TRAF3.

## Bone remodeling

In response to normal wear and tear and mechanical forces and the aging process, bone is continuously remodeled in the adult skeleton by a process in which damaged or effete microscopic portions of bone are removed by osteoclasts and subsequently are replaced by new bone, which is laid down by osteoblasts (3, 18, 19). On trabecular surfaces of spongy (cancellous) bone, bone remodeling units (BRUs) are trench-shaped structures that osteoclasts form by degrading the matrix. They erode to a mean depth of ~60 μm and then tend to work their way along lamellae of collagen, which were laid down previously by osteoblasts, and typically create relatively smooth-bottomed trenches during normal remodeling. The bases of these trenches are marked by the reversal line, a dark line seen in sections stained with H&E and other stains. Osteoclastic resorption is less orderly in pathologic processes in which resorption rates are increased, resulting in reversal lines that are typically irregular and can give the bone a mosaic pattern, seen most classically in Paget's disease of bone (20). Osteoclasts also remodel the more dense cortical bone that encases and protects spongy bone by forming roughly circular tunnels through it. These tunnels are almost completely filled in with new bone to form structures called osteons, which have a small central nutrient artery and vein. This remodeling process involves complex interactions between osteoclastic and osteoblastic cells that couple bone formation to these sites of resorption where coupling factors released from the bone matrix and by osteoclasts attract osteoblast precursors to the site (5).

To initiate bone resorption, osteoclasts first produce hydrochloric acid, which dissolves the mineral in bone, and then they secrete metalloproteases, which breakdown the collagenous matrix (10). Osteoclasts secrete H^+^ ions through protons pumps and Cl^−^ ions pass through chloride channels on the cell membrane on their undersurface adjacent to calcified bone (10, 21). Mutations in the genes involved in matrix demineralization and dissolution account for the majority of human cases of osteopetrosis (3, 10, 21, 22). The osteoclast cell membrane folds to form finger-like processes called the ruffled border that greatly increases the cell surface area for secretion of bone-degrading acid and enzymes (10, 21). Osteoclast cell membranes form a roughly circular tight junction with the bone surface around the ruffled border, called the sealing zone, which effectively creates an enclosed extracellular lysosomal compartment that protects cells in resorption lacunae from the low pH (~5.5) under the cells. The main osteoclast proteolytic enzyme, cathepsin K, functions most effectively at this pH to degrade the matrix after the mineral has been dissolved (10, 21). Degraded matrix particles are passed through the osteoclast cytoplasm to the outer surface of the cell from which they are released into the resorption lacunae (23), where there are nutrient-carrying afferent sinusoids as well as efferent sinusoids that remove these particles to the bloodstream (10). The lacunae appear to be covered by a thin collagenous membrane called a canopy that isolates the lacunae to protect the adjacent bone marrow from the resorptive process (24). Osteoclasts die by apoptosis in the deepest parts of BMUs behind the advancing edges for the resorption lacunae (25, 26), and cytokines released from bone resorption, such as TGFβ1 promote osteoclast apoptosis (27) and attract osteoblast progenitors to the site (28), while macrophages (29) appear to be involved in preparation of the resorbed surface for new bone formation by osteoblasts.

There are estimated to be >1 million BRUs (also called basic multicellular units; BMUs) in the normal adult skeleton, and their numbers increase in many pathologic conditions in response to increased production of cytokines, hormones and growth factors. In many of these conditions, including infections (30, 31), inflammatory/auto-immune diseases (e.g., rheumatoid arthritis) (32), endocrine disorders (20, 33), and metastatic cancers (34, 35) that spread to bone, these factors typically increase bone resorption and inhibit bone formation, leading to generalized bone loss (osteoporosis) or to localized, radiologically lytic lesions.

## Regulation of osteoclast formation and function

Osteoclasts are multinucleated cells that form by fusion of hematopoietic myeloid precursors typically in the bone marrow adjacent to bone surfaces. They can be recognized in H&E-stained sections, and more readily in sections stained histochemically for tartrate-resistant acid phosphatase (TRAP), which osteoclasts secrete (Figure 1). Expression of TRAP is not required for normal bone resorption, but serum TRAP levels correlate positively with the level of skeletal resorption (36). Osteoclast precursors are formed in the bone marrow and are attracted from there into the bloodstream by sphingosine-1 phosphate (S1P) (37), which is produced in large amounts by red blood cells. They are attracted back into the bone marrow to resorption lacunae by RANKL (10, 38) expressed by osteoblastic and immune cells. They are also recruited by CXCL12/SDF1 (39) and by S1P (40), expressed by osteoblastic/stromal cells and osteoclasts, respectively.

**Figure 2:**
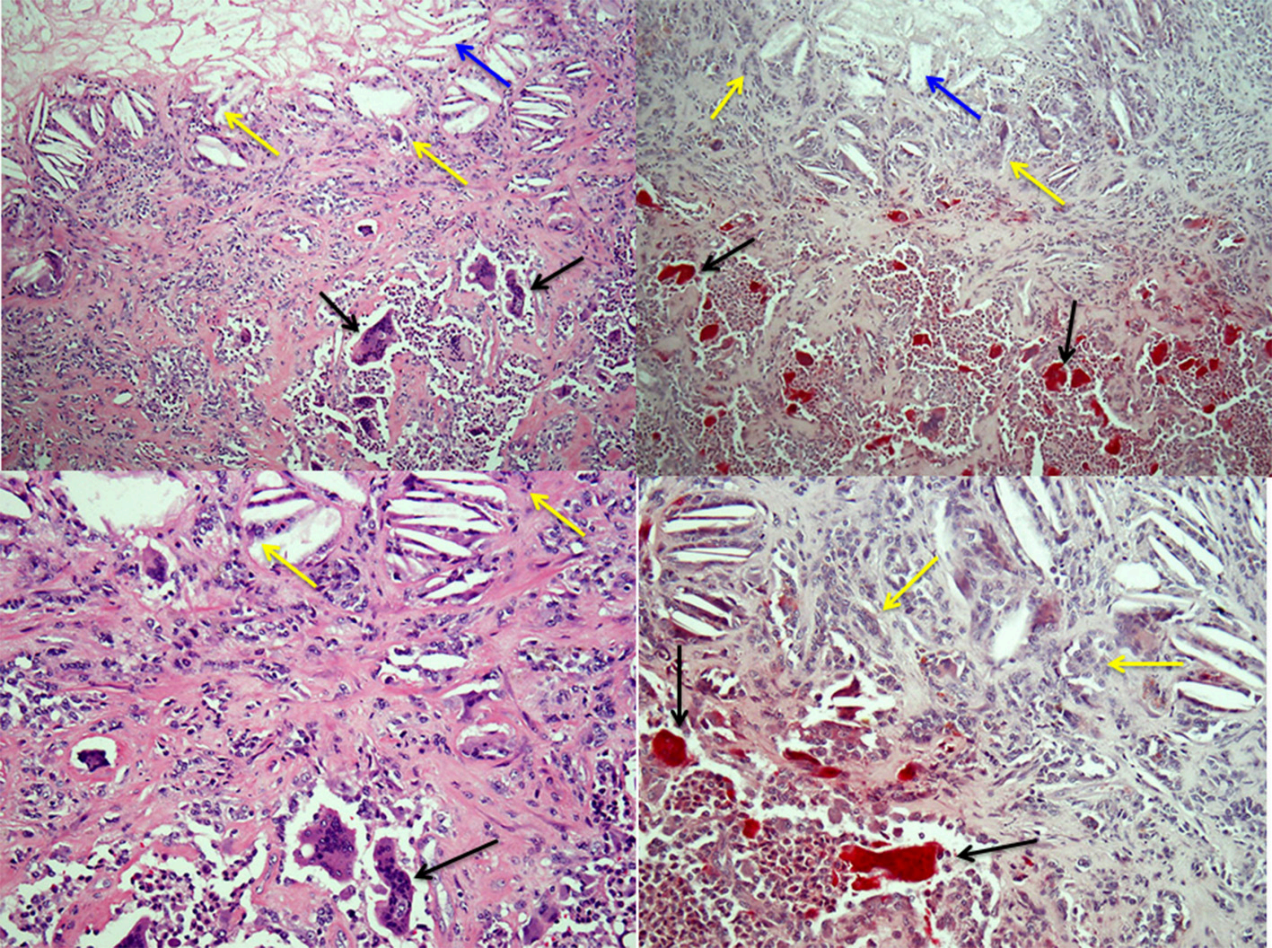
Osteoclasts and multinucleated foreign-body type giant cells in giant cell tumor of tendon sheath. Left-hand panels show H&E-stained sections of a giant cell tumor of tendon sheath with a mix of collagenous stroma, mononuclear cells and osteoclasts (black arrows) in the lower halves of the images and cholesterol clefts (blue arrows) with multinucleated giant cells (yellow arrows) below an area of necrosis in the tumor in the upper halves. The right-hand panels show low and high power images of the lesion with TRAP-positive osteoclasts and TRAP-negative multinucleated giant cells associated with the cholesterol clefts.

Osteoclasts can also form outside the skeleton in a variety of pathologic lesions in humans, including the relatively common giant cell tumor of tendon sheath and the closely related pigmented villonodular tenosynovitis [(41); Figure 2)]. Mesenchymal cells in these soft tissue lesions express RANKL and M-CSF (42), which presumably attract osteoclast precursors from the bloodstream and induce their differentiation into osteoclasts. Osteoclasts can also be observed, sometimes in large numbers, in a small percentage of primary carcinomas (43, 44), including breast, lung, pancreas, and bladder, and in some soft tissue sarcomas, but the molecular mechanisms that induce their formation in these lesions are unknown. Macrophages, like osteoclasts, are derived from myeloid precursors and can comprise up to 40% of the cells in malignant tumors. Tumor cells attract and activate these macrophages (45), which are called tumor-associated macrophages (TAMs). TAMs have multiple functions, some supportive of tumor cell growth and invasion (46), others inhibitory (47). Macrophage/monocytes are also present in benign lesions, including giant cell tumor of tendon sheath and pigmented villonodular tenosynovitis. Some of these cells fuse to form the multinucleated osteoclasts in these lesions, but others can fuse to form TRAP-negative polykaryons [Figure 2; (48)] and these multinucleated cells do not resorb bone. TRAP-negative giant cells can form in numerous other pathologic settings in response to a variety of factors, including cholesterol from dead normal or tumor cells (Figure 2), foreign agents, such as some bacteria and viruses, and surgically implanted graft materials, and their function in these conditions is to degrade them. It is possible that osteoclasts and their mononuclear precursors, like TAMs, have positive or negative influences on the behavior of malignant cells in tumors outside the skeleton, but this has not been studied to date.

M-CSF is expressed by osteoblast lineage cells in the bone marrow and induces expression of RANK by osteoclast precursors, which further differentiate and fuse with one another to form osteoclasts in response to RANKL (5, 10). RANKL is expressed in BMUs in the bone marrow by accessory cells, including osteoblastic/stromal cells (5, 18), B lymphocytes (49) and T lymphocytes (4, 10, 50, 51). RANKL is also expressed and secreted by osteocytes (52, 53), the most abundant cells in bone. Osteocytes start their existence as matrix-forming osteoblasts on bone forming surfaces. Most osteoblasts die by apoptosis when their matrix forming mission has been completed (26), but some of them become embedded within the uncalcified matrix, called osteoid, as it is being formed, and the others remain on the bone surface as flat lining cells. When osteoid becomes mineralized, osteoblasts remain “trapped” and form osteocytes in the calcified bone until they are released during a subsequent remodeling cycle. Osteocytes have numerous dendritic processes that allow them to communicate with each other within the bone and with lining cells on the surfaces of fully calcified bone (54). It is believed that, as a result of this syncytial arrangement, osteocytes can respond to mechanical forces and detect areas of bone that have become damaged and need to be removed by osteoclasts (54).

Osteocytes express both cell membrane-bound and secreted form of RANKL (52). Interestingly, osteocyte-derived RANKL is not required for the formation and activation of osteoclasts that resorb bone during embryonic development in mice, but it is required for normal bone remodeling in the adolescent and adult mouse skeleton (53, 55). RANKL also activates osteoclasts and maintains their survival along with M-CSF in resorption lacunae for up to ~30 days, the average lifespan of osteoclasts. Mice and humans deficient in RANKL, RANK or M-CSF or its receptor *c-fms* develop osteopetrosis (10, 22), which is characterized by failure of removal of mineralized bone matrix from the medullary cavities of long bones and vertebrae during embryonic development (Figure 1). Consequently, osteopetrotic bones are radio-opaque on X-ray and have a typical diagnostic club-shape to their ends because the resorption of cortical bone on the periosteum at metaphyses that gives the ends a concave configuration does not occur. Despite their sclerotic appearance, osteopetrotic bones are weaker than normal bones (22), because the bone formed during development is typically composed of woven, rather than lamellar bone, which is stronger than woven bone.

Osteoblast precursors, like osteoclast precursors, appear to circulate in the blood and are attracted to BMUs by molecules released during bone resorption, including various cytokines, chemokines and growth factors (56), and other osteoclast products, including S1P and collagen fragments (57). Osteoblasts, derived from mesenchymal precursors in the bone marrow, positively and negatively regulate osteoclast formation and activation: osteoblast precursors (presumably at the advancing edges of BMUs) and osteocytes express M-CSF and RANKL to drive and maintain resorption (5, 10). Osteoblast precursors destined to become osteoblasts appear to be attracted to the deeper parts of BMUs after osteoclasts undergo apoptosis. At this site, they form a layer of cells on the lacunar surface and lay down lamellae of bone matrix. Osteoblasts and osteocytes also express osteoprotegerin (OPG), a decoy receptor for RANKL that binds to RANKL and prevents it from binding to RANK to limit osteoclast formation and activation (10, 58). They also express other factors, including Leucine-rich repeat-containing G-protein-coupled receptor 4 (LGR4), a recently identified additional receptor for RANKL (59) that also competes with RANK. LGR4 activates Gα_q_ and GSK3-β signaling, which suppresses expression and activity of NFATc1 (59), a transcription factor required for osteoclast formation (10, 58). The precise details of which subsets of osteoblastic cells promote and inhibit osteoclast formation and where they are located precisely in resorption lacunae remain to be determined. Osteoclasts and their precursors can also positively and negatively regulate osteoblast formation (3, 5), but exactly where these subsets of cells are located in BMUs also remains to be determined.

## The RANKL/RANK/OPG signaling system in osteoclast formation and activation

RANKL expression by osteocytes and accessory cells in bone marrow attracts osteoclast precursors from the bloodstream to resorption lacunae where expression of RANK by precursors is increased in response to M-CSF (60). RANK expression is also induced in osteoclast precursors by the transcription factors, PU.1 and microphthalmia-induced transcription factor (MITF) (61, 62) during the early stages of commitment of these cells to osteoclast differentiation, as well as by IL-34 (63), Wnt5a (64), and TNF (10, 65), which is the major inflammation-inducing cytokine in RA. Many of the accessory cells that express RANKL also express OPG to limit osteoclast formation, and the relative concentrations of these cytokines appear to be a major determinant of the level of bone resorption in normal and disease states (3, 58). A human monoclonal antibody to RANKL has been approved by the FDA for the treatment of a variety of osteolytic bone diseases, including osteoporosis, metastatic bone disease, and multiple myeloma (66, 67).

RANK is expressed by a growing number of cell types in addition to immune cells in bone marrow. These include dendritic cells, which are activated by RANKL expressed by T cells, mammary gland milk-producing cells (58), which fail to develop in RANKL^−/−^ and RANK^−/−^ mice during pregnancy, and consequently mutant mothers are unable to feed their pups. Breast and prostate cancers in humans also express RANK (58), and RANKL/RANK signaling has been implicated in breast cancer metastasis to bone. A few cases of RANK deficiency have been reported in humans (68), but activating mutations in *TNFRSF11A* (the gene encoding RANK) are more common (68). These are associated with early-onset (juvenile) Paget's disease of bone, familial expansile osteolysis and expansile skeletal hyperphosphatasia (69, 70).

OPG is secreted by osteoblasts in response to most of the factors that promote RANKL expression by these cells and in this way it limits osteoclast formation, activity and survival, and the subsequent bone destruction (58). OPG is also expressed by cells in numerous other organs, including the heart, liver, kidney, and spleen, and has been implicated in cardiovascular disease, diabetes, and hypertension (71). Homozygous partial deletions of *TNFRSF11B* (the gene encoding OPG) have been reported in some patients with juvenile Paget's disease, resulting in osteoporosis and increased risk of fractures (72). An inactivating deletion in exon 3 of *TNFRSF11B* is associated with increased bone turnover and deformities of long bones, acetabular protrusion, and kyphosis in some children with idiopathic hyperphosphatasia, an autosomal recessive disease (3, 73).

Wnt/β-catenin signaling regulates osteoblast formation and differentiation from MSCs (74), but it also regulates osteoclast formation. For example, Wnt5a induces RANK expression in osteoclast precursors (64) and canonical Wnt/β-catenin signaling promotes OPG expression by osteoblastic cells (75). In addition, Wnt3a (76) and Wnt16 (75, 77) limit osteoclast formation not only through canonical Wnt signaling, but also through non-canonical signaling by inhibiting RANKL-mediated NF-κB-induced NFATc1 expression. Wnt4a also inhibits osteoclast formation. Wnt4a prevents the formation of a RANKL-induced TRAF6-Tak1-Tab2 complex and instead promotes formation of a Tak1-Tab2-NlK complex, thereby limiting NF-κB p65 nuclear translocation. Through these actions Wnt4a inhibits ovariectomy-induced osteoporosis (76, 78). In addition, activation of β-catenin signaling in early OCPs promotes their differentiation into osteoclasts, but inhibits OC formation in more differentiated precursors (79). Thus, Wnt signaling can have positive and negative regulatory roles in osteoclast formation and activation.

## Roles for TRAFs and NF-κB signaling in osteoclast formation and activation

RANK is a member of the TNF superfamily of receptors, which lack intrinsic protein kinase activity to activate downstream signaling. These receptors recruit a number of proteins to their cytoplasmic domains, including TRAFs, to mediate downstream signaling. In response to RANKL, RANK recruits TRAFs 1, 2, 3, 5, and 6 in OCPs (10, 58); of these, only TRAF6 appears to be necessary for osteoclast formation, since only TRAF6^−/−^ mice are osteopetrotic. Two lines of TRAF6^−/−^ mice were generated independently, and surprisingly one has no OCs, while the other has many osteoclasts that do not resorb bone (10, 58), suggesting that TRAF6 has essential roles in both OC formation and activation. Why these knockout mice have different OC phenotypes has not been explained, but this may reflect different knockout strategies. RANKL/RANK signaling through TRAF6 activates several pathways in OCPs to promote their differentiation and activation. These include NF-κB, c-Jun N-terminal kinase (JNK), c-myc, and phospholipase Cγ/calcineurin/NFATc1 (10, 58).

NF-κB signaling was discovered unexpectedly to be essential for osteoclast formation before the discovery of RANKL or RANK when p50/p52 double knockout were generated. These mice formed no osteoclasts or TRAP-positive mononuclear cells in their bone marrow cavities, which were filled with unremodeled trabecular bone, typical of severe osteopetrosis (80, 81). Subsequent studies showed that the defect in RANKL-induced osteoclast formation from precursor cells in the double knockout mice could be prevented *in vitro* by overexpression of c-fos or NFATc1, indicating that c-fos or NFATc1 acts downstream of NF-κB signaling (82). Other pathways activated by RANKL/RANK/TRAF6 signaling mediate activation of osteoclasts, including Src and mitogen-activated protein kinase kinase 6 (MKK6)/p38/MITF, and to prevent their apoptosis, for example Src and ERK (10, 58).

TRAF2^−/−^ mice die during embryonic development or within 2–3 weeks after birth (83, 84), similar to TRAF3^−/−^ mice (85), making examination of the roles of these TRAFs in skeletal development and in post-natal osteoclast and osteoblast formation challenging. TRAF2^−/−^ and TRAF3^−/−^ mice were reported to have normally formed, but shorter limbs than their WT littermates, suggesting that they were not osteopetrotic. Using fetal liver transplantation as a source of osteoclast precursors from TRAF2^−/−^ mice, another group reported that TRAF2 is required for full TNF-, but not RANKL-induced osteoclastogenesis (86), consistent with TRAF2 having a non-essential function in osteoclastogenesis. Mice deficient in TRAFs 1, 4, and 5 appear to have normal skeletal development (87, 88).

## Roles for TRAF3 in osteoclastic cells

RANKL efficiently processes non-canonical NF-κB protein p100 into p52 and thus induces full osteoclast differentiation. In contrast, TNF does not efficiently process p100 to 52 and this limits osteoclast differentiation (89). Interestingly, TRAF3 protein levels parallel those of p100 during osteoclast differentiation. For example, TNF increases p100 and TRAF3 protein level, associated with limited osteoclast formation, while RANKL induces degradation of TRAF3 protein leading to processing of p100 to p52 and lower p100 levels, associated with increased osteoclast formation (89), consistent with TRAF3 negatively regulating osteoclast formation by preventing p100 processing into p52. Indeed, knockdown of TRAF3 expression promoted TNF induction of osteoclast formation, associated with increased levels of NF-κB inducing kinase (NIK) and enhanced p100 processing to p52 (89). Consistent with this, over-expression of TRAF3 inhibited RANKL-induced osteoclast formation, associated with decreased p100 processing to p52, decreased NIK, RelB and RelA levels as well as a decrease in the osteoclast formation markers, NFATc1 and c-Fos (17). This is consistent with previous studies showing that TRAF3 suppresses both canonical and non-canonical NF-κB signaling (90, 91) and that transgenic mice over-expressing a form of NIK that lacks the TRAF3 binding domain develop osteoporosis due to increased osteoclast formation and activity (92).

Ubiquitination is a common pathway for protein degradation, which can be carried out by proteasomes or lysosome/autophagosomes. Original studies indicated that the proteasome inhibitor, MG-132, did not prevent RANKL-induced TRAF3 degradation (89), but different lysosomal inhibitors, including chloroquine (CQ) and NH4Cl, blocked RANKL-induced degradation of TRAF3 (17). Similarly, the autophagy inhibitors, bafilomycin and 3-Methyladenine, also prevented RANKL-induced TRAF3 degradation. Consistent with this, RANKL promoted TRAF3 co-localization with LAMP2, which CQ blocked (17).

To further explore the role of TRAF3 in osteoclasts, Xiu et al. (17) generated mice with TRAF deleted in osteoclast lineage cells by crossing TRAF3^fl/fl^ mice with lysozyme M^cre^ and cathepsin K^cre^ mice. Lysozyme M targets all myeloid precursor cells, including osteoclast precursors, while cathepsin K targets committed osteoclast precursors and osteoclasts since it is expressed by these cells and is the main metalloproteinase secreted by osteoclasts to dissolve bone matrix. They found that both lines of mice with conditional deletion of TRAF3 had normal skeletal development and phenotype, but they developed early onset osteoporosis due to increased osteoclast formation and activity (17). Treatment of bone marrow macrophages from both lines of transgenic mice with M-CSF and low doses of RANKL resulted in more and larger osteoclasts, which formed earlier than that from wild type littermate mice. These transgenic mice developed more severe bone loss after ovariectomy, but unlike in wild type mice, chloroquine did not prevent ovariectomy-induced bone loss and the associated increased osteoclastogenesis (17). Other investigators have generated these mice using lysozyme M^cre^ mice and reported that 68% of mice aged 15–22-months-old developed various chronic inflammatory lesions, infections or tumors, including B cell lymphomas (93), indicating that TRAF3 in myeloid cells has anti-inflammatory and anti-neoplastic functions. TRAF3 levels decrease in monocytes from humans during aging due to proteasomal degradation (94), and Li et al. have reported that TRAF3 levels decrease in bone in mice during aging (95).

Chloroquine has been used for decades to treat and prevent malaria and is still used in some parts of the world as a first-line anti-inflammatory drug for autoimmune diseases, including RA and systemic lupus erythematosus. Hydroxychloroquine replaced chloroquine in the 1970s and 80s in the US and Europe as an anti-inflammatory drug. Hydroxychloroquine also inhibits bone resorption *in vitro* and *in vivo* (96). Thus, chloroquine or its analogs, including hydroxychloroquine could potentially be used to treat osteoporosis and other osteolytic diseases, particularly if they could be targeted to bone and away from other tissues to reduce side effects, which limit the amount of these drugs that can be administered to patients. To this end, Yao et al. generated bone-targeted conjugates of chloroquine and hydroxychloroquine by linking them to a bisphosphonate, which has high binding affinity for hydroxyapatite, but minimal or no anti-osteoclastic activity (97). Bone-targeted chloroquine more effectively inhibited osteoclast formation and bone resorption *in vitro* and *in vivo* than chloroquine (97). They are currently are testing these *in vitro* and *in vivo* in models of RA and age-related bone loss.

## TRAF3 in TNF-induced osteoclast formation

TNF, like RANKL, induces osteoclast formation by sequentially activating NF-κB/c-fos/NFATc1 signaling (82) and enhancing IκB-α phosphorylation in osteoclast precursors (98). Unlike RANKL, TNF recruits TRAF2, but not TRAF6 to its receptors (86, 99). In fact, TRAF6 appears to negatively regulate TNF-induced canonical NF-κB signaling, based on enhanced TNF-induced expression of IL-6, CXCL1 and GM-CSF in TRAF6-deficient mouse embryonic fibroblasts, associated with enhanced IκB kinase activation and IκB-α degradation (100). We and others have reported that TNF can induce osteoclast formation from WT, RANKL^−/−^, and RANK^−/−^ osteoclast precursors *in vitro* as well as *in vivo* in RANKL^−/−^ and RANK^−/−^ mice when the mice are also deficient in the inhibitory NF-κB protein, p100, which limits osteoclast formation (89, 101). These findings indicate that TNF can induce osteoclast formation independent of RANKL. In contrast, other investigators reported earlier that priming of precursors by RANKL was necessary for TNF induction of osteoclastogenesis (102). This discrepancy may reflect differences in the *in vitro* approaches used by these labs. Despite this controversy, TNF stimulates the expression of RANKL by accessory cells as its major mechanism to indirectly enhance bone resorption, as evidenced by the report that synoviocytes appear to be the major source of RANKL in inflamed joints in RA (14). In contrast to RANKL signaling, which causes TRAF3 degradation, TNF signaling increases protein levels of TRAF3 in osteoclast precursors to limit osteoclast formation (89). As a result, RANKL promotes non-canonical NIK-mediated p100 proteasomal processing to p52, while TNF does not (89). In addition, TNF dose-dependently reduces RANKL-induced osteoclast formation *in vitro* by increasing p100 protein levels in osteoclast precursors (89). Interestingly, RANKL signaling also reduces TNF-induced TRAF3 levels to enhance osteoclast differentiation in the absence of TRAF6 (98). Consistent with this, deletion of TRAF3 in osteoclast progenitor cells enhanced TNF-induced osteoclast formation (98). Thus, TRAF3 and p100 can combine to limit osteoclastogenesis induced by TNF, which also induces expression of other inhibitors of osteoclastogenesis, including IRF-8 and the Notch-induced RPB-Jκ (103).

## Roles for TRAF3 in osteoblastic cells

More recently, the role of TRAF3 has also been investigated in osteoblast progenitors by crossing TRAF3^fl/fl^ mice with Prx1^cre^ mice (95). Prx1 targets mesenchymal progenitor cells, including osteoblastic and chondroblastic cells. These conditional knockout (cKO) mice have normal skeletal development and bone mass until at least 3-months of age, but they develop early onset osteoporosis by 9-months-old through a combination of increased bone resorption and decreased bone formation (95). A role for TRAF3 in mesenchymal cells has not been reported previously, and the mechanisms whereby TRAF3 protects against age-related bone loss are under investigation. However, these recent findings suggest that therapeutic prevention of TRAF3 degradation *in vivo* could increase bone mass in a variety of diseases by preventing bone destruction and promoting bone formation.

## Summary

**Figure 3:**
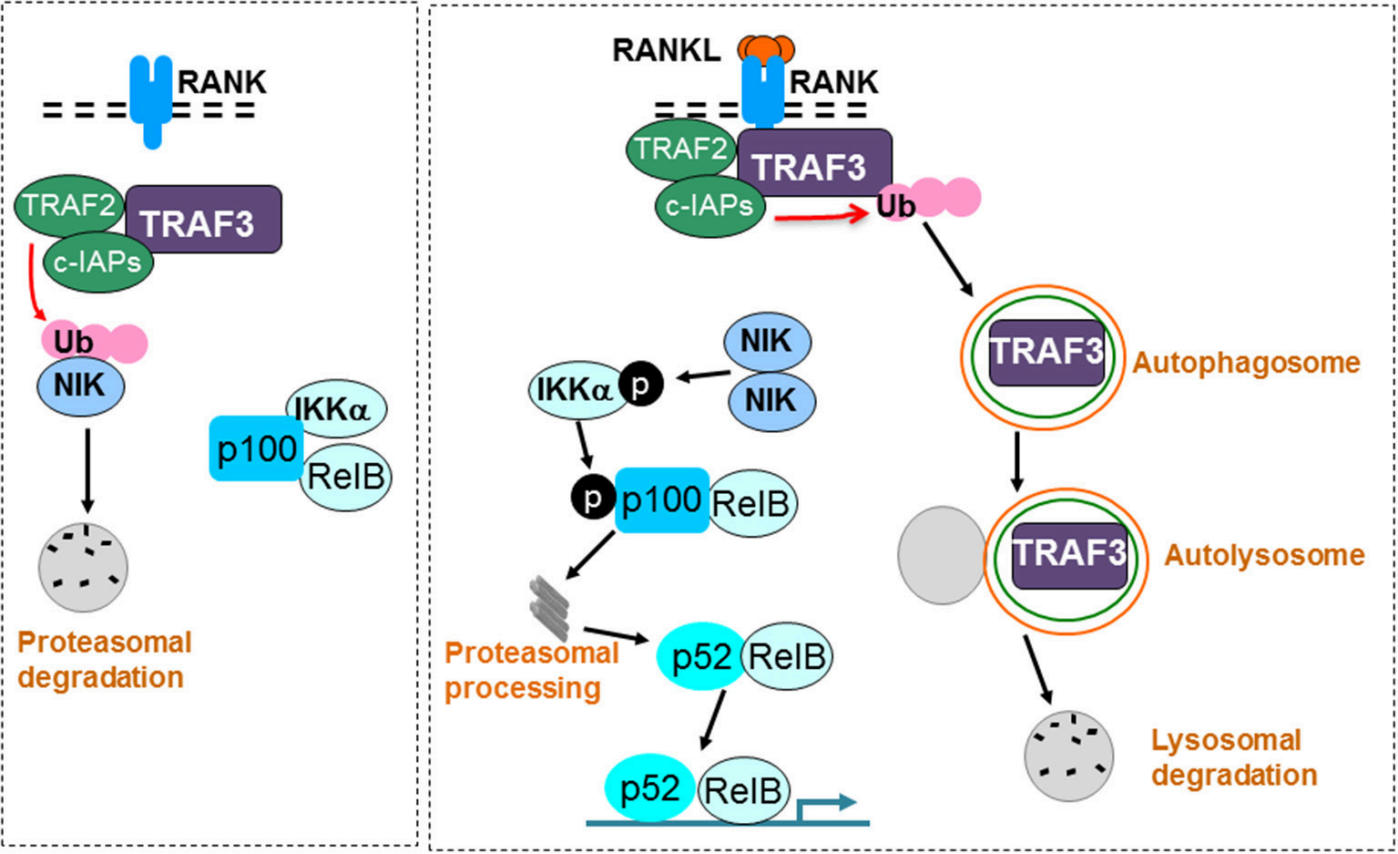
RANKL signaling induces TRAF3 degradation to promote osteoclast formation. A TRAF3/TRAF2/cIAP complex constitutively induces ubiquitination (Ub) and proteasomal degradation of NIK in unstimulated osteoclast precursors. As a consequence, p100 and RelB remain in the cytoplasm in an inactive complex with the inhibitory NF-κB protein, IKKα (left panel). RANKL binding to RANK induces ubiquitination and autophagolysosomal degradation of TRAF3, allowing accumulation of NIK, which phosphorylates and activates IKKα. IKKα then phosphorylates p100, leading to its proteasomal processing to p52. As a result, p52/RelB dimers translocate to the nucleus of osteoclast precursors to promote osteoclast formation (right panel).

**Figure 4:**
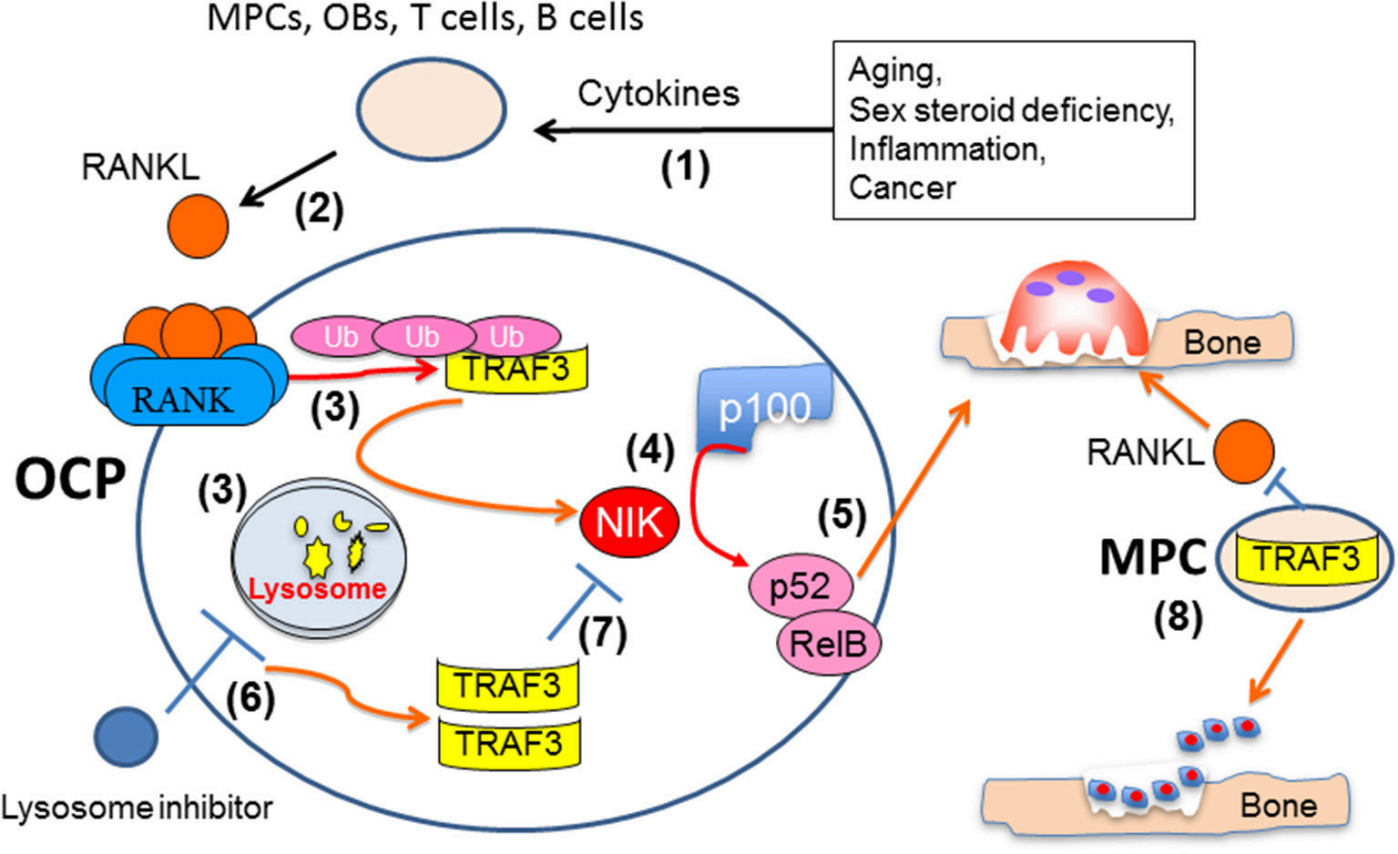
Mechanisms influencing TRAF3 functions and degradation in osteoclast and osteoblast precursors. Multiple pathologic processes and aging can increase production of cytokines, such as TNF, IL-1 and IL-6 (1), to increase production of RANKL by accessory cells, including mesenchymal progenitor (MPCs), osteoblastic (OB), and T and B lymphocytes (2). RANKL binding to RANK in osteoclast precursors results in TRAF3 ubiquitination and lysosomal degradation (3), thus allowing NF-κB-inducing kinase (NIK) to mediate proteasomal processing of p100 to 52 (4) and formation of p52/RelB heterodimers to promote osteoclast formation and bone resorption (5). Inhibitors of lysosomal degradation, such as chloroquine, can prevent degradation of TRAF3, which will promote NIK degradation and inhibit osteoclast formation (7). TRAF3 expression in MPCs also promotes their differentiation into OBs and limits their production of RANKL to maintain bone formation and restrict bone resorption (8).

Normal skeletal development and bone remodeling require the formation and activation of osteoclasts, which are derived from myeloid precursors in the bone marrow. Osteoclasts are formed and activated in response to RANKL, which is expressed by osteoblastic and immune cells in bone. RANKL activates NF-κB signaling in osteoclast precursors by recruiting TRAF6 to its receptor, RANK, and this leads to activation of a number of signaling pathways in these cells that induce osteoclast formation and activation. RANKL signaling also induces autophagosomal degradation of TRAF3 by TRAF2 and cIAPs (Figure 3). This facilitates osteoclast formation by inhibiting TRAF3-induced proteasomal degradation of NIK and promoting p100 processing to p52. Mice with TRAF3 conditionally deleted in osteoclast precursor cells develop early-onset osteoporosis due to increased osteoclast formation. Inhibition of TRAF3 degradation by the autophagosomal inhibitor drug, chloroquine, inhibits osteoclast formation and prevents ovariectomy-induced osteoporosis in mice. The finding that mice with TRAF3 deleted in mesenchymal precursors have increased bone resorption and decreased bone formation, points to TRAF3 having a positive regulatory role in osteoblastic precursors that could be targeted therapeutically to not only inhibit bone resorption, but also stimulate bone formation in common diseases associated with decreased bone mass. These findings suggest that drugs, like chloroquine or cIAP antagonists (104), that inhibit TRAF3 degradation could prevent bone destruction by inhibiting osteoclast formation and stimulating bone formation by enhancing mesenchymal progenitor cell differentiation into osteoblasts in a variety of bone diseases (Figure 4).

## Future directions and gaps in knowledge

Chloroquine or hydroxychloroquine are FDA-approved drugs for the treatment of autoimmune diseases, including rheumatoid arthritis, and may not be ideal drugs to treat age- or menopause-related bone loss because of their known side effects, including blindness that can affect up to 1% of patients, that limit the doses that can be administered (105, 106). Nevertheless, chloroquine and hydroxychloroquine are being studied in clinical trials of patients with multiple myeloma (107, 108) in which they appear to augment the effects of proteasome inhibitors by inducing myeloma cell apoptosis (109). In this setting chloroquine could also potentially inhibit the associated bone resorption and perhaps stimulate new bone formation. One future direction should be attempts to develop small molecule inhibitors that could prevent TRAF2/cIAP-induced TRAF3 degradation. cIAP antagonists have already been developed by a number of pharmaceutical companies as chemotherapeutic agents to promote cancer cell apoptosis with promising results (104) However, one of these cIAP inhibitors appears to also stimulate bone resorption in male, but not female mice, and thus this class of molecules could have detrimental effects on the skeleton of men treated with them as part of a chemotherapeutic regimen for cancer (110).

Findings to date suggest that post-translational modification, rather than increased gene expression, is the major mechanism regulating TRAF3 levels and functions in osteoclast precursors to mediate bone loss in conditions associated with increased bone resorption (17, 89). However, the molecular mechanisms regulating *TRAF3* gene expression in bone cells in normal or pathologic remodeling remain to be determined and could also be a potential target for upregulation in future studies. TRAF3 could also have roles in mature osteoblasts that have yet to be examined.

## Author contributions

All authors listed have made a substantial, direct and intellectual contribution to the work, and approved it for publication.

### Conflict of interest statement

The authors declare that the research was conducted in the absence of any commercial or financial relationships that could be construed as a potential conflict of interest. The reviewer BH and handling Editor declared their shared affiliation at the time of the review.
